# Supplementary material for: Molecular memory with downstream logic processing exemplified by switchable and self-indicating guest capture and release
Source: Nat Commun. 2019 Jan 21;10:49. doi: 10.1038/s41467-018-07902-7 (PMC6341106; doi:10.1038/s41467-018-07902-7)
Supplement: Supplementary file 1 — Supplementary Information [file 41467_2018_7902_MOESM1_ESM.pdf]

Supplementary Information

Molecular Memory with Downstream Logic Processing exemplified by Switchable and Self-indicating Guest Capture and Release

Daly et al

Supplementary Methods

1. Synthesis and characterization data of all compounds used in the study

Synthesis of 1,7,21,27-tetraoxa-[7.1.7.1]-paracyclophane (1).
4,4'-Dihydroxybenzophenone **5** (3.20 g, 14.9 mmol) and diiodopentane (4.86 g, 15.0 mmol) were dissolved in acetone (400 ml, freshly distilled HPLC grade, dried over anhydrous potassium carbonate) and slowly added, with the aid of a dropping funnel whose capillary tip is dipping below the surface, to a refluxing suspension of acetone (600 ml) and cesium carbonate (8.0 g, 25 mmol) over 12 hours. The colourless solution turned a pale yellow colour. This mixture was then refluxed for a further three days. The hot reaction mixture was then filtered through a silica/hyflo plug and the filtrate chilled to 4 °C whereupon a white solid precipitated out. The solid was purified using flash silica chromatography eluting with dichloromethane: ethyl acetate (95:5 v/v) yielding the desired white plate-like solid (0.8 g, 10%).
Melting Point = 187-188 °C. Elemental analysis; Found: C, 74.46; H, 6.02. Required for $C_{36}H_{36}O_6.H_2O$: C, 74.22; H, 6.53. ^1H NMR ($CDCl_3$, 500 MHz): δ7.68(d, 8H, ArH, J=8.7Hz), 6.83(d, 8H, ArH, J=8.7Hz), 4.10(t, 8H, O-CH_2, J=6.0 Hz), 1.87(m, 8H, O-CH_2-CH_2, J=6.0, 6.6 Hz), 1.72(t, 4H, O-$(CH_2)_2$-CH_2, J=6.6 Hz). ^{13}C NMR ($CDCl_3$, 101 MHz) δ194.31, 162.03, 132.08, 130.64, 114.12, 67.53, 27.63, 21.72. i.r. (ν_{max})(KBr): 3447, 2943, 2878, 1642, 1602, 1576, 1510, 1467, 1433, 1419, 1317, 1304, 1287, 1262, 1167, 1150, 1115, 1074, 1040, 1014, 963, 947, 928, 847, 835, 768, 684, 626, 614, 512 cm^{-1}. u.v. λ_{max} (ε_{max})(MeOH): 289 nm (64000 M^{-1} cm^{-1}). m/z(%), (E.I.): 564(61, M^+), 552(4), 509(3), 368(4), 283(9), 227(6), 215(7), 197(5), 185(6), 157(4), 139(4), 121(53), 83(100), 69(35), 59(24).

Synthesis of 9,17,29,37-Tetraiodo-14,34-Dioxo-1,17,21,27-Tetraoxa[1.7.1.7]-paracyclophane (6).
1 (0.30 g, 0.53 mmol) was dissolved in hot chloroform (25 ml, 0.31 mol), dried over anhydrous potassium carbonate) and to this solution was added silver trifluoroacetate (1.00 g, 4.9 mmol). The reaction vessel was sealed with a stopper and stirred vigorously for four days. The resultant mixture was then refluxed with chloroform (400 ml) for one hour and filtered hot through a hyflo supercel plug and the filtrate was collected. The yellow silver residue present on the hyflo was washed several times with hot chloroform. The combined filtrate was evaporated to dryness to give a black solid. The solid was then treated with hot ethanol (200 ml) and refluxed for one hour, cooled and filtered. The resultant off-white solid was then washed with cold ethanol and dried (0.35 g, 92%).
Melting Point = 282-283 °C. Elemental analysis; Found: C, 38.34; H, 2.81. Required for $C_{36}H_{32}O_6I_4.3H_2O$: C, 38.50; H, 3.38. ^1H NMR ($CDCl_3$, 500 MHz): δ8.10(s, 4H, ArH), 7.69(d, 4H, ArH, J=8.7 Hz), 6.79(d, 4H, ArH, J=8.6 Hz), 4.23(t, 8H, O-CH_2, J=5.6 Hz), 1.98(m, 8H, O-CH_2-CH_2, J=6.0, 6.6 Hz), 1.85(t, 4H, O-$(CH_2)_2$-CH_2, J=6.6 Hz). ^{13}C NMR (DMSO, 101 MHz) δ178.64, 160.85, 140.85, 132.04, 131.79, 112.76, 87.06, 69.80, 28.13, 22.43. i.r. (ν_{max})(KBr): 3441, 2948, 2376, 2346, 1647, 1586, 1550, 1485, 1473, 1438, 1382, 1303,

1264, 1244, 1218, 1145, 1079, 1050, 1035, 1003, 981, 963, 909, 898, 820, 758, 706, 686, 663, 625, 541 cm^{-1}. u.v. λ_{max} (ε_{max})(MeOH): 285 nm (38000 M^{-1} cm^{-1}). m/z(%), (E.I.): 1068(26, M^+), 941(9), 815(3), 591(9), 564(44), 368(32), 280(26), 246(31), 203(100).

Synthesis of 9,17,29,37-Tetracarboxy-14,34-Dioxo-1,7,21,27-Tetraoxa-[7.1.7.1.]-paracyclophane (3).

6 (300 mg, 0.0280 mmol) and tetrakis(triphenylphosphine)-Palladium-(0) (150 mg, 0.130 mmol) were suspended in dimethylformamide (45 ml) in a three necked round bottomed flask (100 ml). Potassium carbonate (3.0 g, 22 mmol) dissolved in water (6 ml) was added and stirred. A carbon monoxide balloon was attached via a tap outlet. The reaction vessel was degassed using another tap outlet and a water pump. Once the system was fully degassed, carbon monoxide was passed through the system until saturation. At this point the balloon tap was opened and the reaction mixture stirred at 60 °C overnight. The mixture was cooled, water (90 ml) added and the aqueous layer washed with dichloromethane (3x30 ml). The aqueous layer was then acidified using dilute hydrochloric acid. This produced a brown solid, which was filtered and dried (0.11 g, 48%).

Melting Point = >300 °C. Elemental analysis; Found: C, 62.82; H, 5.00. Required for $C_{40}H_{36}O_{14}.H_2O$: C, 63.32; H, 5.01. ^{1}H NMR (D_2O/NaOD, 300MHz): δ7.58(s, 4H, Ar$\underline{H}$), 7.18(d, 4H, Ar$\underline{H}$, J=8.8 Hz), 6.69(d, 4H, Ar$\underline{H}$, J=8.8 Hz), 4.02(t, 8H, O-C$\underline{H}_2$, J=5.6 Hz), 1.72(m, 8H, O-CH_2-C$\underline{H}_2$, J=5.6, 5.4 Hz), 1.52(t, 4H, O-$(CH_2)_2$-C$\underline{H}_2$, J=5.4 Hz). ^{13}C NMR (DMSO, 101 MHz) δ192.32, 167.04, 160.78, 135.07, 132.83, 129.06, 122.14, 113.41, 68.76, 27.29, 21.50. i.r. (ν_{max})(KBr): 3854, 3433, 2940, 2375, 2346, 1734, 1653, 1602, 1574, 1497, 1476, 1420, 1384, 1265, 1156, 1085, 1039, 986, 934, 833, 762, 669, 649, 626, 519 cm^{-1}. u.v. λ_{max} (ε_{max})(MeOH:H_2O)(1:1, v/v), pH 10.0: 304 nm (31000 M^{-1} cm^{-1}). m/z(%), (E.I.): 741(100, M + H^+), 724(8), 697(12), 651(4), 398(3), 317(8), 301(9).

Synthesis of 9,17,29,37-Tetracarboxy-14,34-Dihydroxy-1,7,21,27-Tetraoxa[7.1.7.1]-paracyclophane (4).

3 (0.070 g, 0.094 mmol) was dissolved in water (5.0 ml, 280 mmol) with the aid of a few drops of dilute sodium hydroxide and then sodium borohydride (0.10 g, 2.6 mmol) added and stirred overnight at room temperature (ca. 20 ^{o}C). A few drops of acetic acid were then added to destroy any unreacted sodium borohydride. The mixture was then acidified to approximately pH 1 with dilute hydrochloric acid. The precipitated product was then filtered, washed with water and dried (0.04 g, 57%).

Melting Point = >300 °C. Elemental analysis; Found: C, 62.82; H, 5.00. Required for $C_{40}H_{40}O_{14}.H_2O$: C, 62.99; H, 5.51. ^{1}H NMR (D_2O/NaOD, 300MHz): δ7.45(s, 4H, Ar$\underline{H}$), 7.03(d, 4H, Ar$\underline{H}$, J=7.4 Hz), 6.92(d, 4H, Ar$\underline{H}$, J=7.2 Hz), 5.77(d, 2H, -C$\underline{H}$-OH), 4.06(b, 8H, O-C$\underline{H}_2$), 1.74(b, 8H, O-CH_2-C$\underline{H}_2$), 1.54(b, 4H, O-$(CH_2)_2$-C$\underline{H}_2$). ^{13}C NMR (DMSO, 101 MHz) δ168.00, 156.58, 137.90, 130.68, 128.47, 113.71, 97.10, 70.53, 68.70, 28.26, 22.09. i.r. (ν_{max})(KBr): 3855, 3433, 3249, 2944, 2374, 2345, 1719, 1611, 1582, 1497, 1471, 1433, 1399, 1243, 1188, 1156, 1084, 1041, 1006, 920, 825, 780, 730, 669, 655, 618 cm^{-1}. u.v. λ_{max} (ε_{max})(MeOH:H_2O)(1:1, v/v), pH 10.0: 285 nm

(3900 M^{-1} cm^{-1}). m/z(%), (E.I.): 744(100, M^+), 728(4), 701(17), 655(4), 402(3), 321(5), 305(9).

Synthesis of 14,34-Dihydroxy-1,7,21,27-Tetraoxa[7.1.7.1]-paracyclophane (2).

A suspension of sodium borohydride (100 mg, 2.64 mmol) in ethanol (5 ml) was added dropwise to a stirring solution of **1** (50 mg, 0.089 mmol) dissolved in tetrahydrofuran (10 ml) and stirred overnight. A few drops of acetic acid and water (20 ml) was added to destroy the reducing agent and the mixture extracted with 3x15 ml of dichloromethane. The organic layer was dried with sodium sulfate and evaporated to dryness to yield the desired white solid (25 mg, 50%).

Melting point = >300 °C. m/z, (E.S.) found: 591.2723 (M+Na^+). Calculated for $C_{36}H_{40}O_6Na$: 591.2721. ^{1}H NMR ($CDCl_3$, 500MHz): δ7.19(d, 8H, Ar$\underline{H}$, J=8.7 Hz), 6.77(d, 8H, Ar$\underline{H}$, J=8.7 Hz), 5.72(d, 2H, -C$\underline{H}$OH, J=5.6 Hz), 3.95(t, 8H, OC$\underline{H}_2$, J=8.1 Hz), 1.78(m, 8H, $OCH_2C\underline{H}_2$), 1.57(m, 4H, $O(CH_2)_2C\underline{H}_2$). ^{13}C NMR ($CDCl_3$, 101 MHz) δ158.37, 136.51, 127.35, 114.44, 75.47, 67.80, 28.68, 22.87. i.r. (ν_{max})(KBr): 3365, 2361, 1469, 1348, 1279, 1130, 1002, 946, 827, 709, 617 cm^{-1}. u.v. λ_{max} (ε_{max})(MeOH): 283 nm (8000 M^{-1} cm^{-1}). m/z(%), (E.I.): 591(30, M+Na^+), 551(15), 497(14), 448(25), 394(16), 343(16), 340(17), 301(32), 289(87), 235(100).

Synthesis of Trimethyl-4-[(1,1,1-trimethylammonio)methyl]benzyl ammonium dibromide (7)[1]

α,α'-Dibromo-*p*-xylene (0.50 g, 1.9 mmol) was dissolved in dimethyl-formamide (50 ml). Trimethylamine (0.6 ml, 3.8 mmol) was then added slowly with stirring. After 10 min a white precipitate formed and the suspension was stirred for a further 20 hours. The precipitate was then filtered and dried yielding a white solid (0.71 g, 98%).

Melting point = 349-350 °C (lit.[1] >320 °C).^{1}H NMR (D_2O, 500MHz): δ7.46(s, 4H, Ar$\underline{H}$), 4.32(s, 4H, ArC$\underline{H}_2$), 2.88(s, 18H, $N^+C\underline{H}_3$). ^{13}C NMR (DMSO, 101 MHz) δ133.66, 130.79, 67.57, 52.32. i.r. (ν_{max}) KBr: 3433, 3003, 2964, 2796, 2361, 1635, 1481, 1429, 1398, 370, 1233, 1219, 1113, 991, 969, 919, 899, 850, 791, 738, 567 cm^{-1}. m/z(%), (E.I.): 407, 405, 403(6,M^++Na^+), 303, 301(100,M^+-Br^-), 284(10), 267(8), 244(12), 214(20).

Synthesis of Triethyl-4-[(1,1,1-triethylammonio)methyl]benzylammonium dibromide (8)[2]

α,α'-Dibromo-*p*-xylene (1.0 g, 3.8 mmol) was dissolved in dimethylformamide (70 ml). Triethylamine (1.0 ml, 7.6 mmol) was then added slowly with stirring. After 30 min a white precipitate formed and the suspension was stirred for a further 20 hours. The precipitate was then filtered and dried yielding a white solid (0.56 g, 32%).

Melting point = 251-252 °C. ^{1}H NMR (D_2O, 500MHz): δ7.71(s, 4H, Ar$\underline{H}$), 4.54 (s, 4H, ArC$\underline{H}_2$), 3.32(q, 12H, $N^+C\underline{H}_2$, J=7.2 Hz), 1.47(t, 18H, $N^+CH_2C\underline{H}_3$, J=7.2 Hz). ^{13}C NMR (DMSO, 101 MHz) δ133.03, 129.73, 58.69, 52.07, 7.55. i.r. (ν_{max}) KBr: 3433, 3010, 2968, 2361, 2340, 1650, 1470, 1386, 1182, 1148, 1009, 812, 794, 575 cm^{-1}. m/z(%), (E.I.): 491, 489, 487 (4,M^++ Na^+), 387, 385(78,M^+-Br^-), 368(10), 277(20), 250(28), 222(100).

Synthesis of 1-[4-(1-azoniabicyclo[2.2.2]oct-1-ylmethyl)benzyl]-1-azoniabicyclo[2.2.2] octane dibromide (9)

α,α'-Dibromo-*p*-xylene (1.19 g, 4.5 mmol) was dissolved in dimethyl-formamide (70 ml). Quinuclidine (1.0 g, 9.0 mmol) was then added slowly with stirring. After 60 min a white precipitate formed and the suspension was stirred for a further 20 hours. The precipitate was then filtered and dried yielding a white solid (0.71 g, 98%).

Melting point = 385-386 °C. Elemental analysis; Found: C, 50.20; H, 8.04; N, 5.32. Required for $C_{22}H_{34}N_2Br_2.2H_2O$: C, 50.47; H, 7.26; N, 5.35. ^{1}H NMR (D_2O) 500MHz: δ7.67(s, 4H, Ar<u>H</u>), 4.45(s, 4H, ArC<u>H</u>$_2$), 3.48(t, 12H, N^+C<u>H</u>$_2$, J=7.9Hz), 1.76 (bm, 12H, N^+ (C<u>H</u>$_2$CH$_2$)$_3$CH), 2.22(bm, 2H, N^+(CH$_2$CH$_2$)$_3$ C<u>H</u>), 1.99 (bm, 12H, N^+(CH$_2$C<u>H</u>$_2$)$_3$CH). ^{13}C NMR (DMSO, 101 MHz) δ133.40, 129.48, 65.50, 53.74, 23.33, 19.45. i.r. (ν_{max}) KBr: 3433, 3365, 2955, 2881, 2361, 2085, 1654, 1610, 1464, 1385, 1226, 1076, 836, 609, 573 cm^{-1}. m/z(%), (E.I.): 511, 509, 507(7,M^++Na^+), 457(16), 407, 405 (100,M^+-Br^-), 388(10), 371(8), 214(22).

2. Additional transformations and stability tests of the molecular logic arrays

Oxidation of 14,34-Dihydroxy-1,7,21,27-tetraoxa-[7.1.7.1]-paracyclophane (2) with air in DMSO

14,34-Dihydroxy-1,7,21,27-tetraoxa-[7.1.7.1]-paracyclophane (50 mg, 0.09 mmol) was suspended in DMSO (3 ml) and cesium hydroxide (ca. 1 mg, 0.007 mmol) was added. A reflux condenser was attached and the mixture heated to 90 ^{o}C overnight. Then the solvent was removed under vacuum and the residue dissolved in chloroform (100 ml) and washed with water (3x20 ml). The organic layer was then dried with sodium sulfate, filtered and concentrated to approximately 10 ml. This was placed in a conical flask and the solvent allowed to evaporate on the bench to give a white crystalline solid. ^{1}H-NMR and TLC analysis confirmed that the ketone, 1,7,21,27-tetraoxa-[7.1.7.1]-paracyclophane (**1**), had been regenerated.

Oxidation of 9,17,29,37-Tetracarboxy-14,34-dihydroxy-1,7,21,27-tetraoxa[7.1.7.1]-paracyclophane (4) with air in DMSO

9,17,29,37-Tetracarboxy-14,34-dihydroxy-1,7,21,27-tetraoxa[7.1.7.1]-paracyclophane (50 mg, 0.07 mmol) was added to DMSO (5 ml) in a two neck flask and caesium hydroxide (approx. 1 mg, 0.007 mmol) added. An efficient double walled reflux condenser was attached and the other neck was sealed with a rubber septum, a needle was placed though this septum so that its point was just below the surface of the liquid and its end open to the air. A vacuum was attached to the top of the reflux condenser and arranged so that air was drawn through the needle and into the solution. The mixture was then heated at 90 ^{o}C overnight. Then the DMSO was removed under reduced pressure and the residue dissolved in water. The solution was acidified with 4M HCl and a precipitate formed. This was centrifuged, the supernatant was discarded, more distilled water was added and the process repeated. The pellet was then flushed from the centrifuge vial with distilled water and dried under reduced pressure at 60 ^{o}C to give a white solid. ^{1}H-NMR and TLC

analysis confirmed that the ketone, 9,17,29,37-Tetracarboxy-1,7,21,27-tetraoxa-[7.1.7.1]-paracyclophane (**3**), had been regenerated.

Oxidation of 9,17,29,37-Tetracarboxy-14,34-dihydroxy-1,7,21,27-tetraoxa[7.1.7.1]-paracyclophane (4) with $KMnO_4$ in water

9,17,29,37-Tetracarboxy-14,34-dihydroxy-1,7,21,27-tetraoxa[7.1.7.1]-paracyclophane (50 mg, 0.07 mmol) was suspended in distilled water (100 ml) and sonicated. Aqueous 5M NaOH solution was added until the solution was clear (ca. 2.5 ml). $KMnO_4$ (100 mg, 0.67 mmol) was then added and the mixture was kept at 40 ^oC for 5 hours with vigorous stirring. After this time, methanol (5 ml) was added and kept until the purple color had faded. The solution was filtered while hot and the methanol removed under reduced pressure. The solution was made upto 100 ml with water before acidification with 5M HCl until a precipitate formed. This was centrifuged, the supernatant was decanted, more distilled water was added and the process repeated. The pellet was then flushed from the centrifuge vial with distilled water and dried under reduced pressure at 60 ^oC to give a white solid. ^1H-NMR analysis confirmed that the ketone, 9,17,29,37-Tetracarboxy-1,7,21,27-tetraoxa-[7.1.7.1]-paracyclophane (**3**), had been regenerated.

Stability test of 7 under the oxidation conditions which were applied to 4 with $KMnO_4$ in water

7 (200 mg, 0.90 mmol) was dissolved in distilled water (50 ml). $KMnO_4$ (142 mg, 0.90 mmol) was then added and the mixture was kept at 40 ^oC for 5 hours with vigorous stirring. After this time, methanol (10 ml) was added and kept until the purple color had faded. The solution was filtered, hydrobromic acid added and evaporated to dryness under reduced pressure. ^1H-NMR analysis confirmed that **7** is the sole component.

Stability test of 7 under the reduction conditions which were applied to 3 with $NaBH_4$ in water

7 (200 mg, 0.90 mmol) was dissolved in water (10 ml), sodium borohydride (1.02 g, 27 mmol) added and stirred for 24 hours at room temperature (ca. 20 ^oC). A few drops of hydrobromic acid were then added to destroy unreacted sodium borohydride and evaporated to dryness under reduced pressure. ^1H-NMR analysis confirmed that **7** is the sole component.

3. Complexation-induced shifts in ^1H NMR spectra of 7-9 with 4

All spectra are obtained at 25 ^oC, after annealing the samples at 60 ^oC for 20 min.

Supplementary Table 1. Chemical shift values for the binding of **7** with **4**.

Proton	**7**	**4**+**7** (*Δδ*)[a]
ArH	7.72	6.98 (-0.74)
BzH	4.58	4.22 (-0.36)
αH	3.14	2.85 (-0.29)

a. (-) = upfield shifts.

Supplementary Table 2. Chemical shift values for the binding of **8** with **4**.

Proton	**8**	**4**+**8** (*Δδ*)[a]
ArH	7.66	7.08 (-0.58)
BzH	4.50	4.24 (-0.26)
αH	3.27	3.05 (-0.22)
βH	1.42	1.31 (-0.11)

a. (-) = upfield shifts.

Supplementary Table 3. Chemical shift values for the binding of **9** with **4**.

Proton	**9**	**4**+**9** (*Δδ*)[a]
ArH	7.62	7.10 (-0.52)
BzH	4.40	4.09 (-0.31)
αH	3.46	3.04 (-0.42)
βH	2.19	2.03 (-0.16)
γH	1.97	1.71 (-0.26)

a. (-)= upfield shifts.

4. Ultraviolet absorption spectra

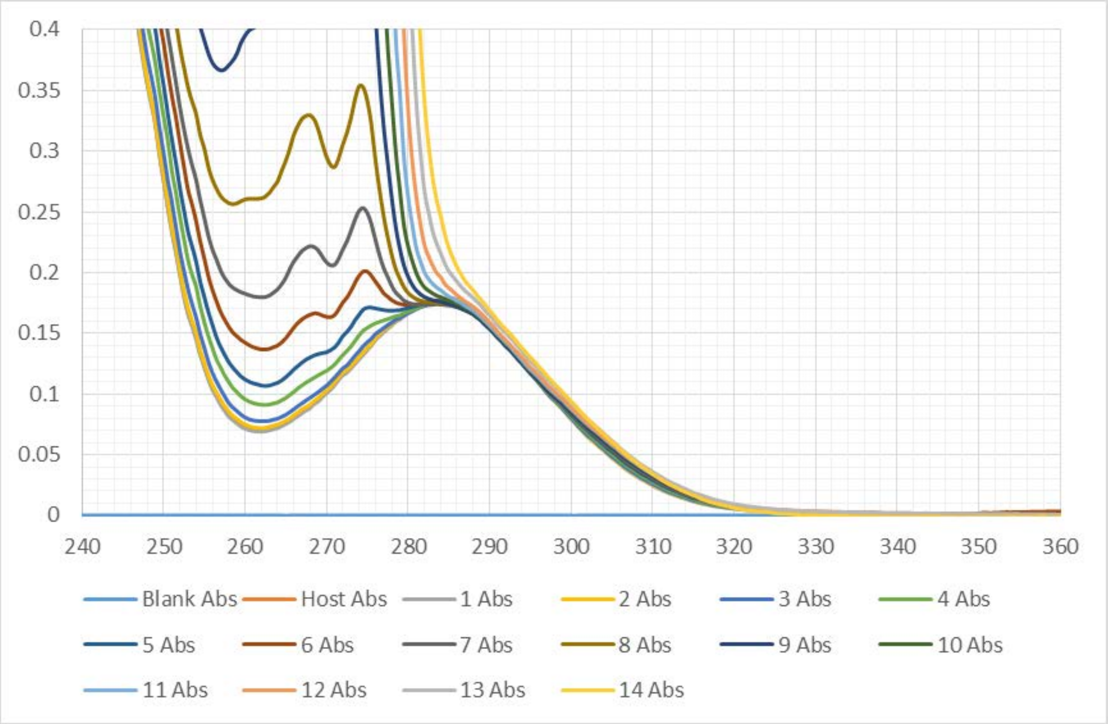

Supplementary Figure 1. Ultraviolet absorption spectra of 5.5x10^{-5} M **4** in water at pH 10.0, in the presence of **7**. The spectra are essentially constant in the region largely free of guest interference (>285 nm and at lower guest concentrations). The p**7** values used are; ∞, 6.01, 5.51, 5.01, 4.51, 4.26, 4.01, 3.76, 3.51, 3.26, 3.01, 2.76, 2.51 and 2.01.

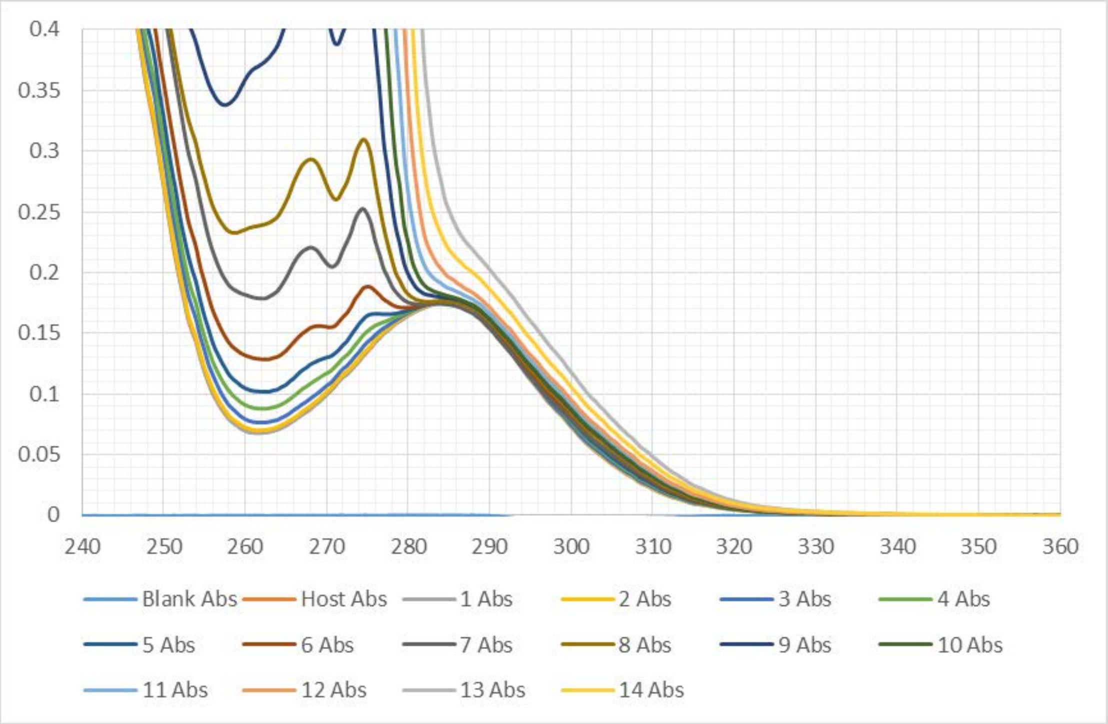

Supplementary Figure 2. Ultraviolet absorption spectra of 5.5x10^{-5} M **4** in water at pH 10.0, in the presence of **8**. The spectra are essentially constant in

the region largely free of guest interference (>285 nm and at lower guest concentrations). The p**7** values used are; ∞, 6.01, 5.51, 5.01, 4.51, 4.26, 4.01, 3.76, 3.51, 3.26, 3.01, 2.76, 2.51 and 2.01.

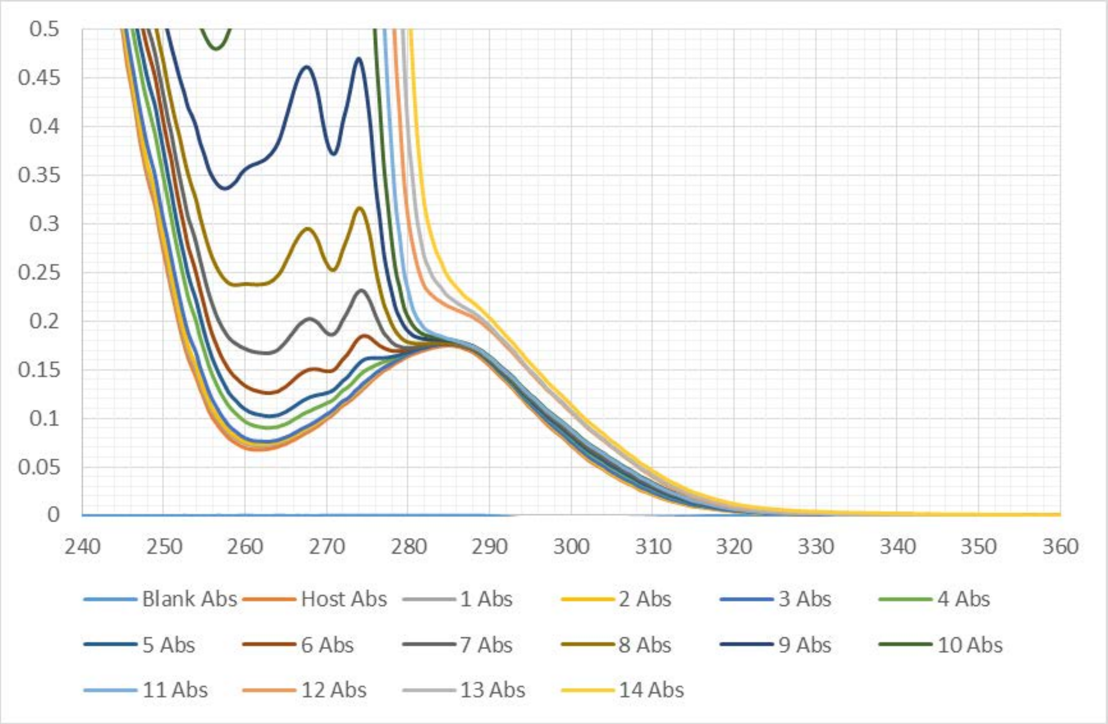

Supplementary Figure 3. Ultraviolet absorption spectra of $5.5x10^{-5}$ M **4** in water at pH 10.0, in the presence of **9**. The spectra are essentially constant in the region largely free of guest interference (>285 nm and at lower guest concentrations). The p**7** values used are; ∞, 6.01, 5.51, 5.01, 4.51, 4.26, 4.01, 3.76, 3.51, 3.26, 3.01, 2.76, 2.51 and 2.01.

5. Fluorescence quenching data

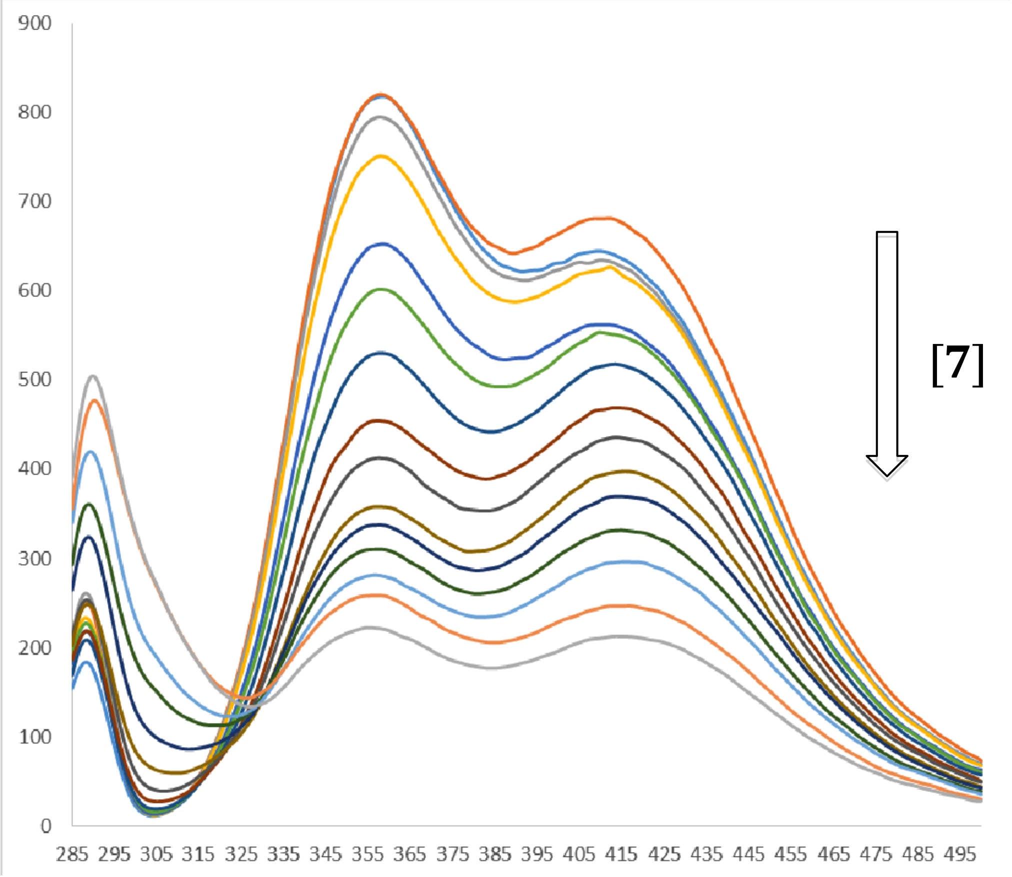

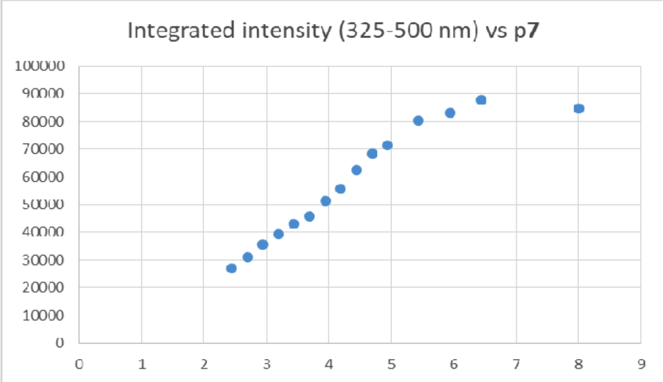

Supplementary Figure 4. Fluorescence spectra [intensity (I_F) - wavelength (nm) plots] of 2.5x10^{-5} M **4** in water at pH 10.0, excited at 290 nm, in the presence of **7**. Spectra in order of decreasing intensity at 356 nm have p**7** values of ∞, 6.44, 5.94, 5.44, 4.94, 4.69, 4.44, 4.19, 3.94, 3.69, 3.44, 3.19, 2.94, 2.69 and 2.44. The Rayleigh scatter peak at 290 nm is also shown. The plot of integrated intensity versus p**7** is shown immediately below.

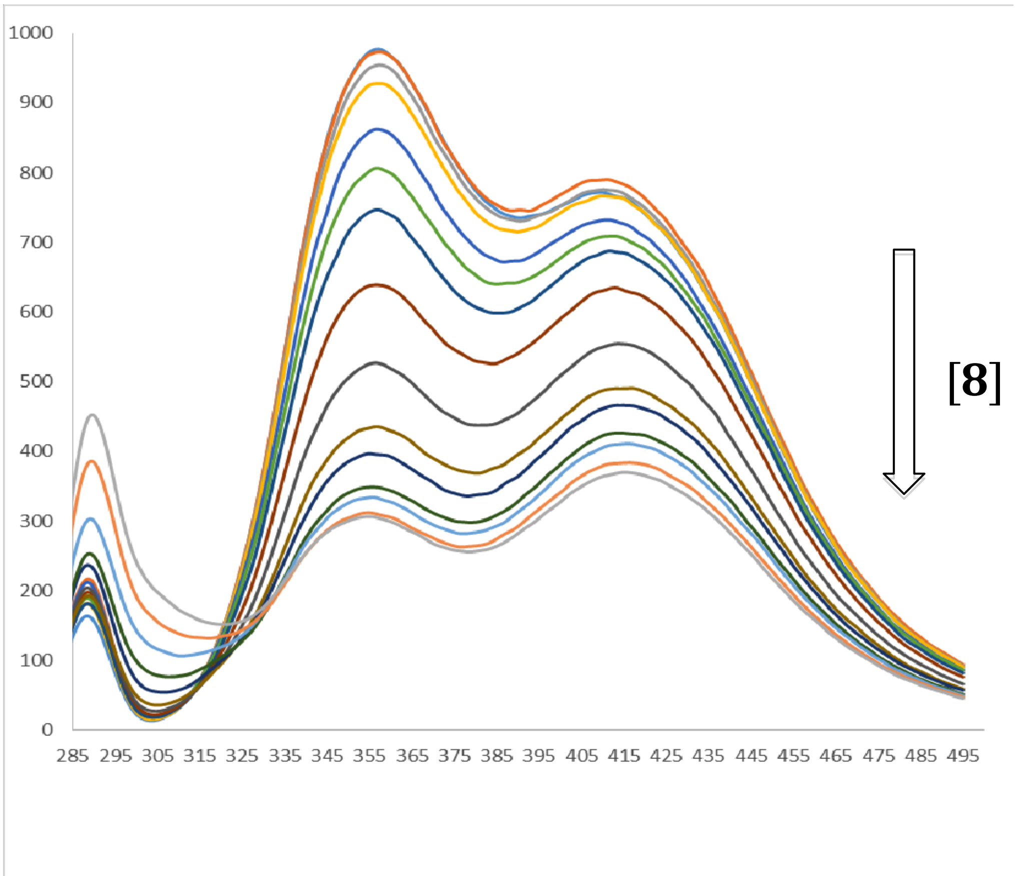

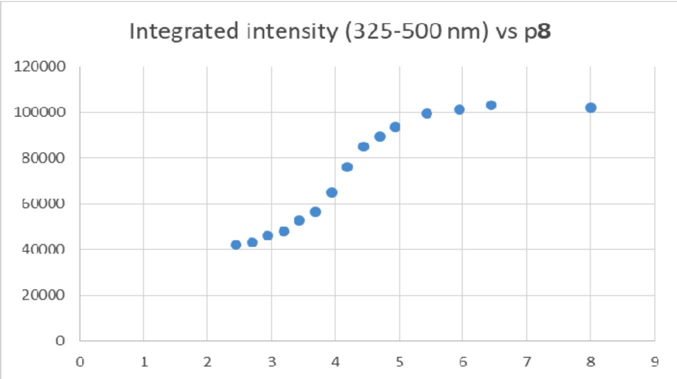

Supplementary Figure 5. Fluorescence spectra [intensity (I_F) - wavelength (nm) plots] of 2.5x10^{-5} M **4** in water at pH 10.0, excited at 290 nm, in the presence of **8**. Spectra in order of decreasing intensity at 356 nm have p**8** values of ∞, 6.44, 5.94, 5.44, 4.94, 4.69, 4.44, 4.19, 3.94, 3.69, 3.44, 3.19, 2.94, 2.69 and 2.44. The Rayleigh scatter peak at 290 nm is also shown. The plot of integrated intensity versus p**8** is shown immediately below.

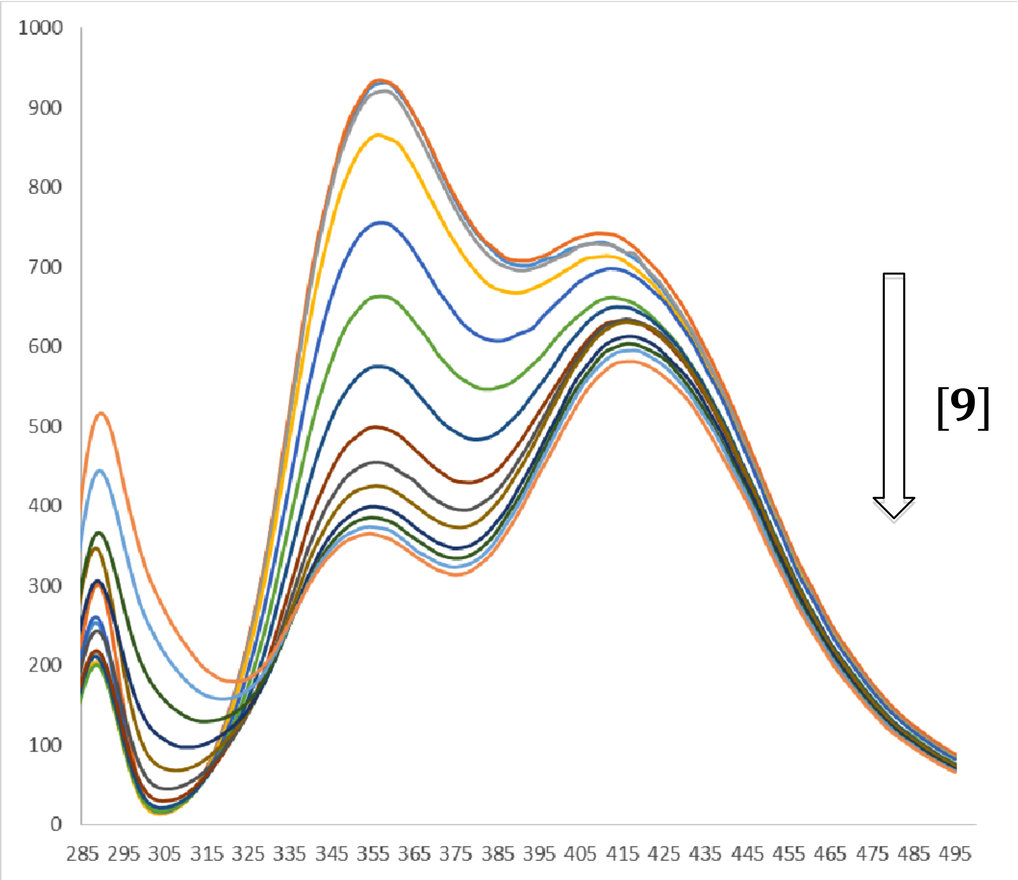

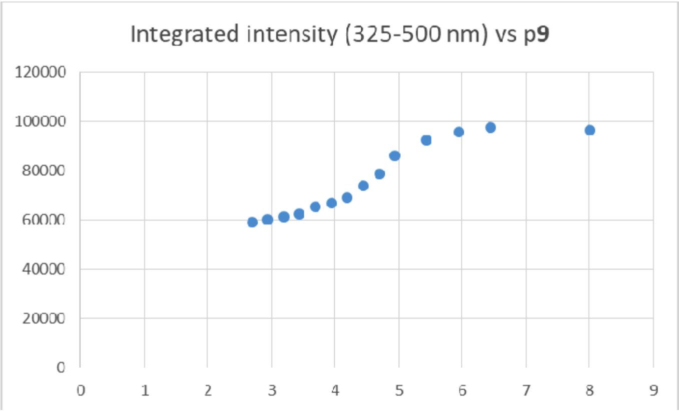

Supplementary Figure 6. Fluorescence spectra [intensity (I_F) - wavelength (nm) plots] of 2.5x10^{-5} M **4** in water at pH 10.0, excited at 290 nm, in the presence of **9**. Spectra in order of decreasing intensity at 356 nm have p**9** values of ∞, 6.44, 5.94, 5.44, 4.94, 4.69, 4.44, 4.19, 3.94, 3.69, 3.44, 3.19, 2.94, 2.69 and 2.44. The Rayleigh scatter peak at 290 nm is also shown. The plot of integrated intensity versus p**9** is shown immediately below.

6. ΔG_{PET} calculation for the quenching of fluorescence of 4 by 7

The Weller equation, as given by Grabowski and Dobkowski[3] is;

$\Delta G_{PET} = - E_{S\ \mathrm{for}\ \mathbf{4}} + E_{ox\ \mathrm{for}\ \mathbf{4}} - E_{red\ \mathrm{for}\ \mathbf{7}} - 0.1$

where the last term (in eV) is the ion-pairing energy in a polar medium.

E_S = hc/λ, where the energies of the absorption maximum wavelength for **4** (285 nm) and the fluorescence maximum wavelength for **4** (356 nm) are averaged. This gives $E_{S\ for\ \mathbf{4}}$ = 3.9 eV.
$E_{ox\ for\ \mathbf{4}}$ is approximated by $E_{ox\ for\ \mathbf{2}}$ which is measured as +1.7 V (vs sce, MeCN, Bu_4NBF_4, 60 °C used for adequate solubility).
$E_{red\ for\ \mathbf{7}}$ is measured as -2.1 V (vs sce, DMF, Bu_4NBF_4).
So, ΔG_{PET} is calculated to be -0.2 eV. Thus PET is thermodynamically possible for the quenching of fluorescence of **4** by **7**.

E_{red} values for **8** and **9** are measured as -2.0 V and -2.1 V respectively (vs sce, DMF, Bu_4NBF_4). So the ΔG_{PET} values are similarly calculated to be -0.3 and -0.2 eV respectively. PET is thermodynamically possible for the quenching of fluorescence of **4** by **8** and by **9** too.

7. X-ray crystallographic Data

Structure of 14,34-Dioxo-1,7,21,27-tetraoxa-[7.1.7.1]-paracyclophane (1)

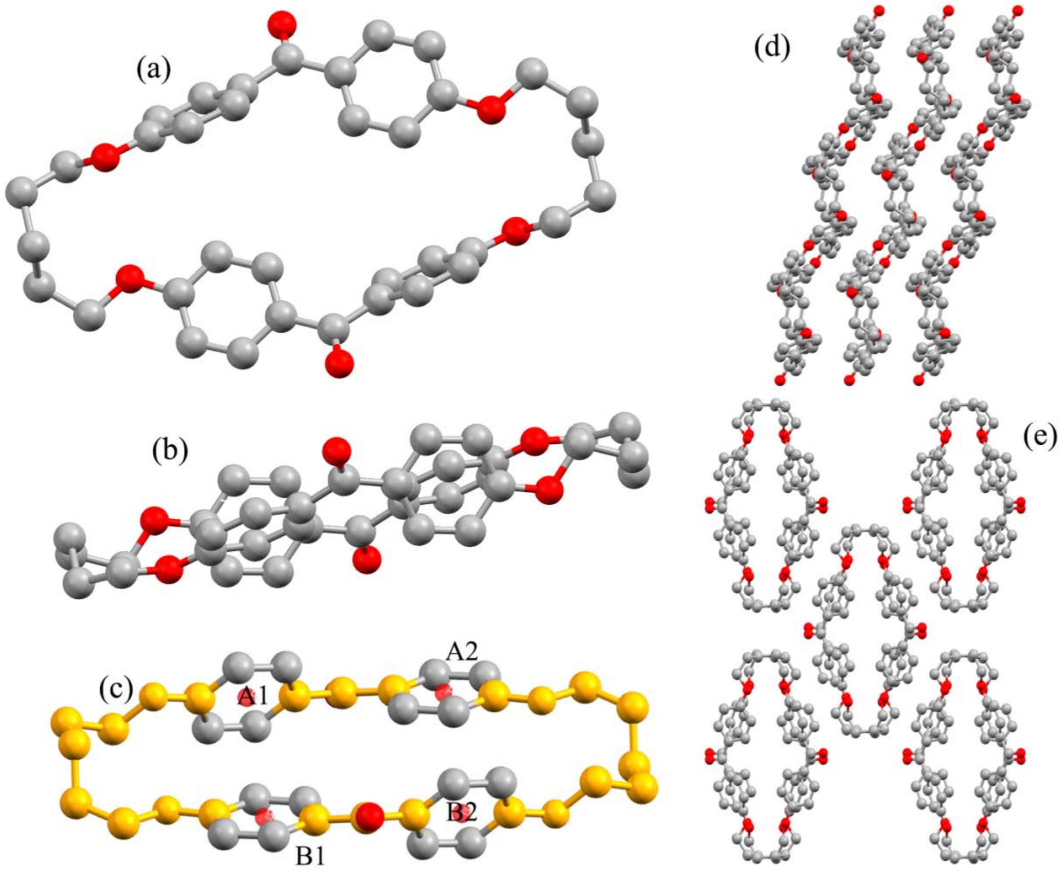

Supplementary Figure 7. Crystal structure obtained for **1**. (a) Plan view, (b) side elevation view, (c) twisted view. Legend indicates ring naming; atoms shown in orange are used to define the molecular mean macrocycle plane. (d) and (e) Side elevation and plan view of packing order respectively.

Crystal data for $C_{36}H_{36}O_6$ (1) (M = 564.65 g/mol): monoclinic, space group C2/c (no. 15), a = 17.0438(12) Å, b = 18.5687(13) Å, c = 9.5106(7) Å, β = 106.4400(17)°, V = 2886.9(4) Å^3, Z = 4, T = 100 K, μ(MoKα) = 0.087 mm^{-1}, $Dcalc$ = 1.299 g/cm^3, 23446 reflections measured (4.38° ≤ 2Θ ≤ 54.98°), 3300 unique (R_{int} = 0.0982, R_{sigma} = 0.0457) which were used in all calculations. The final R_1 was 0.0497 (>2sigma(I)) and wR_2 was 0.1495 (all data).

Supplementary Table 4. Angles determined from crystal structure of **1**.

Number	Object1	Object2	Angle
1	Carbonyl 1	1b	21.49
2	Carbonyl 1	1a	39.00
3	Carbonyl 1	Macro	38.11
4	Carbonyl 2	Macro	38.11
5	Carbonyl 2	2b	39.00
6	Carbonyl 2	2a	21.49
7	1b	1a	58.2
8	Macro	1a	75.85
9	Macro	1b	17.67
10	Macro	2b	75.85
11	Macro	2a	17.67

Supplementary Table 5. Distances determined from crystal structure of **1**.

Number	Object 1	Object 2	Length
1	1a	2a	5.104
2	1b	2b	5.104
3	1a	1b	5.17
4	2a	2b	5.17
5	1a	2b	7.359
6	2a	1b	7.17

The angles taken from the crystal structure show that the phenylene rings can be divided into two pairs using the relative angle of each to the macrocycle mean plane. Rings 1a and 2b have angles to the plane at 75.85^o and these rings are said to be erect. The outermost carbon of these phenylene rings with respect to the macrocycle mean plane has a perpendicular distance of 1.277 Å. Rings 1b and 2a are said to have collapsed, these have an angle of 39.00^o to the mean plane of the macrocycle. The outermost carbon of these

rings has a perpendicular distance of 0.254 Å. The centroid to centroid distance shows that there is an intra-annular edge to face interaction between opposite phenylene rings i.e. from 1a to 2a and from 2b to 1b. The molecular volume of the molecule was calculated using Olex2 and was found to be 481.27 Å^3 with and error of 0.143%.

Structure of 14,34-Dihydroxy-1,7,21,27-tetraoxa-[7.1.7.1]-paracyclophane (2)

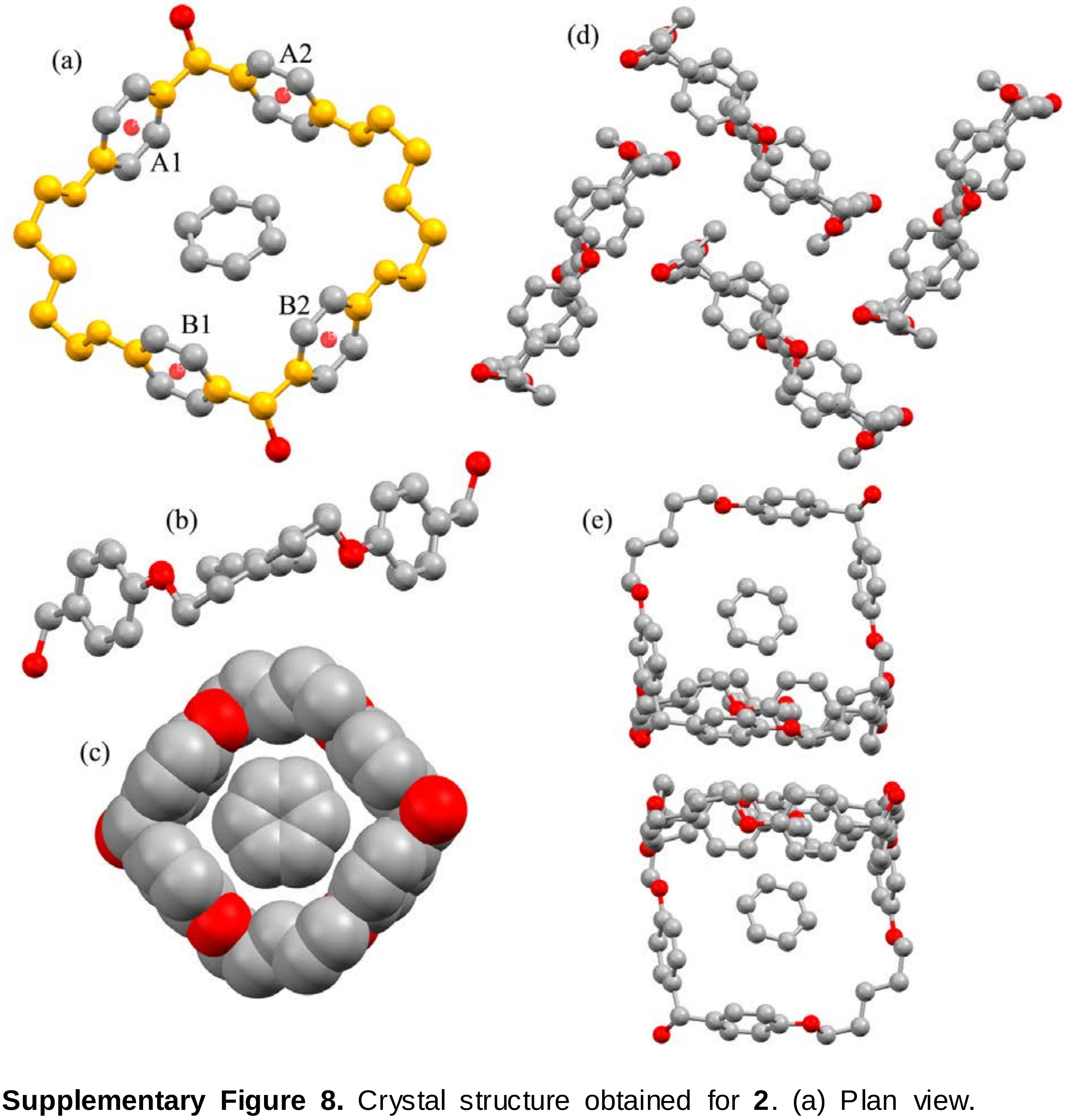

Supplementary Figure 8. Crystal structure obtained for **2**. (a) Plan view. Legend indicates ring numbering – Orange atoms are those that are used to calculate mean macrocycle plane. (b) Side elevation view, (c) plan view in space fill representation. (d) and (e) Side elevation and plan view showing packing order respectively.

Crystal data for $C_{42}H_{46}O_6$ (2) (*M* =646.79 g/mol): monoclinic, space group $P2_1/c$ (no. 14), a = 13.7636(9) Å, b = 8.4906(6) Å, c = 15.6271(11) Å, β =

107.530(4)°, V = 1741.4(2) Å^3, Z = 2, T = 100 K, μ(MoKα) = 0.081 mm^{-1}, $Dcalc$ = 1.234 g/cm^3, 21663 reflections measured (5.42° ≤ 2Θ ≤ 55°), 3981 unique (R_{int} = 0.1281, R_{sigma} = 0.0770) which were used in all calculations. The final R_1 was 0.0653 (>2sigma(I)) and wR_2 was 0.1746 (all data).

Supplementary Table 6. Angles determined from crystal structure of **2**.

Number	Object1	Object2	Angle
1	Macrocycle mean plane	A1	82.97
2	Macrocycle mean plane	A2	87.81
3	Macrocycle mean plane	B1	87.81
4	Macrocycle mean plane	B2	82.97
5	Macrocycle mean plane	Benzene	7.82
6	A1	A2	79.93
7	B1	B2	79.93
8	A1	B2	0.00
9	B1	A2	0.00

Supplementary Table 7. Distances determined from crystal structure of **2**.

Number	Object1	Object2	Length
1	A1	A2	4.63
2	A1	B1	9.28
3	A1	B2	10.34
4	A2	B1	10.41
5	A2	B2	9.28
6	B1	B2	4.63
7	Benzene	A1	5.17
8	Benzene	A2	5.21
9	Benzene	B1	5.21
10	Benzene	B2	5.17

The absence of a carbonyl and its electronic effects mean that the phenylene rings are free to rotate so that the angle between each and the macrocycle mean plane is > 80° in all instances, and thus all of the walls are said to be ‘erect’. The perpendicular distance from the macrocycle mean plane to the outermost carbon of each of the phenylene rings is now more uniform with distances from 0.990 to 1.384 Å. The rotation of the rings means that the opposing rings (A1-B2 and A2-B1) are perpendicular; however the expansion of the distances between these rings means that no intra-annular interactions are possible.

Benzene is found exclusively in the intramolecular cavity and no disorder is observed in its position, this lack of disorder can be explained by edge to face contacts between the benzene ring and each of the phenylene rings of the

host, as measurements show the distance from the benzene centroid to that of each ring in the macrocycle is ca 5.2 Å. The molecular volume was measured using Olex2 and found to be 563.660 Å^3 (error = 0.147%) this represents an increase in volume of over 17% through oxidation of the bridging carbonyl alone.

Comparison of crystal structure of 14,34-Dioxo-1,7,21,27-tetraoxa-[7.1.7.1.]-paracyclophane (1) to known structures

4,4'-dimethoxybenzophenone, has been previously crystallized by Norment and Karle[4] and subsequently by Fun, Franklin, Jebas and Balasubramanian,[5] although both groups crystallized different polymorphs the structures are broadly similar and measurements of angles and distances found in these structures indicate the angles adopted by the neighbouring phenyl rings are dominated by electronic rather than steric effects.

Monoclinic polymorph of 4,4'-dimethoxybenzophenone[4]

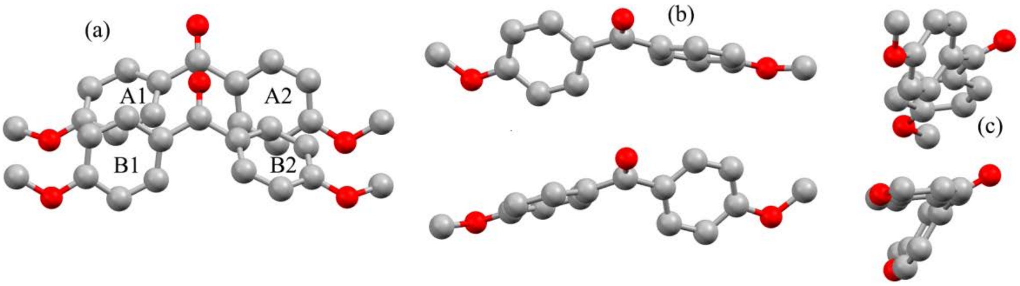

Supplementary Figure 9. Crystal Structure obtained by Norment and Karle for 4-4'-dimethoxybenzophenone, (a) Side elevation view with ring numbering, (b) plan view, (c) second side elevation view.

Supplementary Table 8. Relevant angles taken from Norment and Karle's 4,4'-dimethoxybenzophenone crystal data.

Number	Object1	Object2	Angle
1	Carbonyl 1	A1	35.61
2	Carbonyl 1	A2	23.36
3	A1	A2	54.07
4	Carbonyl 2	B1	25.68
5	Carbonyl 2	B2	35.05
6	B1	B2	55.06

Supplementary Table 9. Relevant Distances taken from Norment and Karle's 4,4'-dimethoxybenzophenone crystal data.

Number	Object1	Object2	Length
1	A1	B1	4.802
2	A2	B2	4.775
3	A1	A2	5.103
4	B1	B2	5.087
5	B1	A2	6.999
6	A1	B2	6.986

Triclinic polymorph of 4,4'-dimethoxybenzophenone[5]

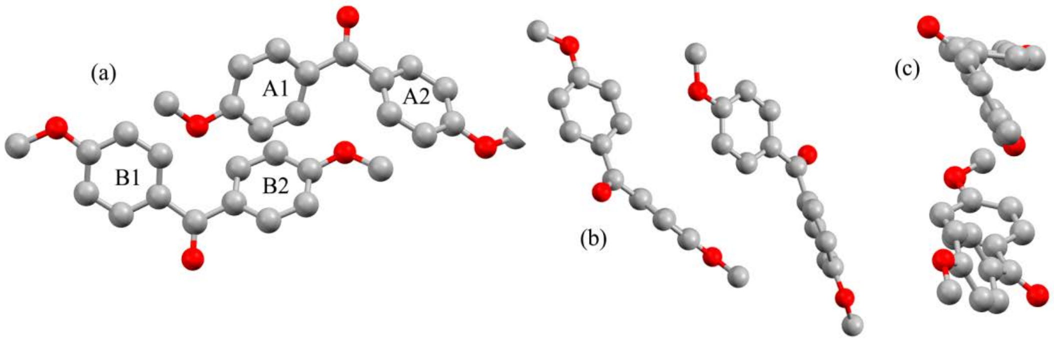

Supplementary Figure 10. Crystal Structure obtained by Fun *et al.* for 4,4'-dimethoxybenzophenone, (a) Side elevation view with ring numbering, (b) Plan view, (c) second side elevation view.

Supplementary Table 10. Relevant angles taken from Fun *et al.* 4,4'-dimethoxybenzophenone crystal data.

Number	Object1	Object2	Angle
1	Carbonyl 1	A1	21.37
2	Carbonyl 1	A2	35.49
3	A1	A2	52.12
4	Carbonyl 2	B1	26.1
5	Carbonyl 2	B2	35.35
6	B1	2	55.72

Supplementary Table 11. Relevant distances taken from Fun *et al.* 4,4'-dimethoxybenzophenone crystal data.

Number	Object1	Object2	Length
1	A1	B1	6.17
2	A2	B2	6.156
3	A1	A2	5.09
4	B1	B2	5.069
5	A1	B2	10.245
6	A2	B1	4.756

2,7,11,16-Tetraoxa-9,18-dioxo-1,8,10,17(1,4)tetrabenzenaoctadecaphane[6]

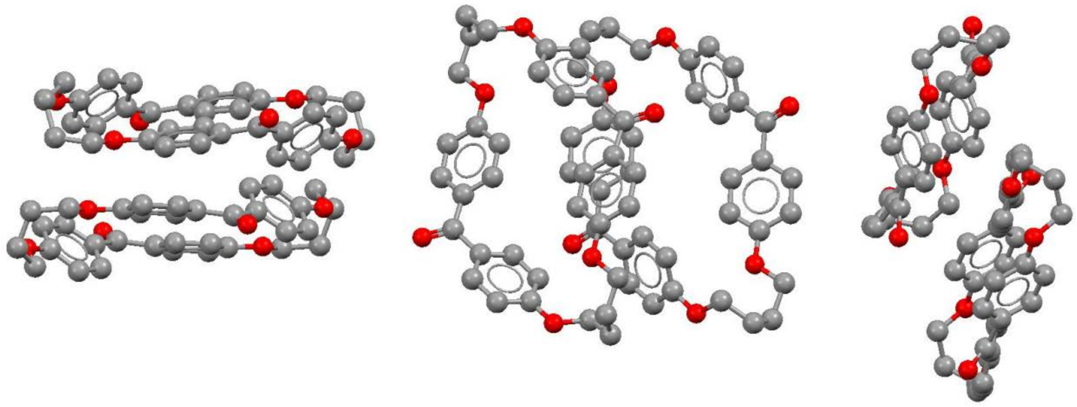

Supplementary Figure 11. Representation of crystal structure obtained by Kuś and Jones for 2,7,11,16-Tetraoxa-9,18-dioxo-1,8,10,17(1,4)tetrabenzenaoctadecaphane.

Like the two examples above this 4C bridged paracyclophane contains two molecules within the unit cell each with slightly different geometries as shown in the figure above. For ease of analysis the molecules have been analysed in isolation the rings are numbered as before but have been given a prefix either 'T' for top molecule or 'B' for bottom molecule.

Supplementary References

1. Phillips, A. P. Synthetic hypotensive agents. II. Some hexamethylene-1,6-bis-t-amines and bis-quaternary salts as ganglionic blocking agents. *J. Am. Chem. Soc.* **77**, 1693–1695 (1955).

2. Robinson, D. B., Rognlien, J. L., Bauer, C. A. & Simmons, B. A. Dependence of amine-accelerated silicate condensation on amine structure. *J. Mater. Chem*. **17**, 2113–2119 (2007).

3. Grabowski, Z. R. & Dobkowski, J. Twisted intramolecular charge transfer (TICT) excited states: Energy and molecular structure. J. *Pure Appl. Chem*. **55**, 245-252 (1983).

4. Norment, H. G. & Karle, I. L. The crystal structures of deoxyanisoin and *p*,*p*'-dimethoxybenzophenone. *Acta Crystallogr*. **15**, 873-878 (1962).
5. Fun, H. K., Franklin, S., Jebas, S. R. & Balasubramanian, T. 4,4 '-dimethoxybenzophenone: a triclinic polymorph. *Acta Crystallogr*. **E64**, O1256-U1845 (2008).
6. Kuś, P. & Jones, P. G. Synthesis of new tetraoxacyclophanes containing benzophenone units. *Polish J. Chem*. **74**, 965-979 (2000).
